# Chemotactic behavior of *Campylobacter fetus* subspecies towards cervical mucus, bovine placenta and selected substances and ion

**DOI:** 10.1590/1984-3143-AR2021-0008

**Published:** 2021-08-06

**Authors:** Dionei Joaquim Haas, Jonata de Melo Barbieri, Elaine Maria Seles Dorneles, Andrey Pereira Lage

**Affiliations:** 1 Departamento de Medicina Veterinária Preventiva, Escola de Veterinária, Universidade Federal de Minas Gerais, Belo Horizonte, MG, Brasil; 2 Departamento de Medicina Veterinária, Universidade Federal de Lavras, Lavras, MG, Brasil

**Keywords:** bacterial chemotaxis, chemoattractants, tissue tropism, bovine genital tract, bovine genital campylobacteriosis

## Abstract

The chemotaxis of *C. fetus* subsp. *venerealis* and *C. fetus* subsp. *fetus* was determined in the presence of bovine cervical mucus and bovine placental extract. Some reported substances and ion in those materials, such amino acids, ferrous iron, hormones, sugars and organic acids were also investigated. Bovine cervical mucus, bovine placenta extracts and some substances and ion of these materials namely L–fucose, L– aspartate, L–glutamate, L–serine, ferrous iron, fumarate, pyruvate and succinate were chemoattractants. The chemottraction was significantly larger in higher concentrations of the tested substances and ion and significant differences among tested strains were also observed. Meso-erythritol and hormones bovine placental lactogen, 17β-estradiol, and progesterone did not elicit chemotactical response. In conclusion, this chemotactic behavior may guide the *C. fetus* navigation in the bovine host's genital tract and be an important cofactor of tissue tropism for this bacterium.

## Introduction

*C. fetus* subsp. *venerealis* and *C. fetus* subsp. *fetus* are important cause of reproductive disorders in cattle. *C. fetus* subsp. *fetus* causes sporadic abortions and *C. fetus* subsp. *venerealis* the bovine genital campylobacteriosis (BGC), a venereal disease that causes early embryonic death, resulting in high rates of return to estrous, at longer and irregular cycles, and, to a lesser frequency, abortions (Alves et al., 2011; Sahin et al., 2017; Balzan et al., 2020; Haas et al., 2020). Therefore, large economic losses occur due to decrease production of milk and calves, increase calving interval, and large number of open cows at the end of the breeding season (McCool et al., 1988; Pellegrin et al., 2002).

Despite this great impact on animal health, the mechanisms involved in the pathogenesis of infection of the two *C. fetus* subspecies in cattle, especially regarding to tissue tropism, remains elusive. The clinical signs observed suggest that *C. fetus* subsp. *venerealis* has a strong tropism for bovine genital tract, reaching the bovine uterus via ascending route, while *C. fetus* subsp. *fetus* shows marked tropism to uterus during pregnancy, reaching the placenta by translocation from its intestinal habitat (Vargas et al., 2002; Sahin et al., 2017; Silveira et al., 2018; Farace et al., 2019; Balzan et al., 2020). Therefore, it has been speculated that tissue tropism of *C. fetus* subspecies is dictated by the presence of local substrates, as amino acids, organic acids, sugars, iron and hormones (Walsh et al., 1973; Ware, 1980; Mshelia et al., 2007; Giobergia et al., 2019; Balzan et al., 2020), which could be potentially attractants to bacteria and chemotaxis-guide to bacterial movements.

Chemotaxis enables bacteria to move according to chemical gradients, which allows them to adapt better to their natural habitats via moving toward favorable conditions and away from hostile surroundings, affords key physiological benefits, including enhanced access to growth substrates (Porter et al., 2011; Bi and Sourjik, 2018; Korolik, 2019). Another important implication of chemotaxis is that it also plays an important role in infection and disease, since it enables bacteria to find suitable colonization sites and maintain their preferred niches (Matilla and Krell, 2018; Yang and Ottemann, 2019). As this is required for optimal host infection and pathogenicity, chemotaxis have been shown to be important for the initiation of the several diseases (Porter et al., 2011; Johnson and Ottemann, 2018; Matilla and Krell, 2018), including those caused by *Campylobacter* species, as *C. jejuni* (Korolik, 2019). For *C. jejuni* infection, chemotaxis is an important prerequisite for host colonization and pathogenesis, which could also be linked to niche specificity (Lertsethtakarn et al., 2011; Chandrashekhar et al., 2017; Korolik, 2019).

Orthologues proteins of the chemotaxis signal transduction pathway has been observed in *C. fetus* (Fahmy et al., 2012), including *C. fetus* subsp. *fetus* ATCC 27374^T^ (NCTC 10842^T^); and *C. fetus* subsp. *venerealis* ATCC 19438^T^ (NCTC 10354^T^) type strains (Stynen et al., 2011; Oliveira et al., 2016). The chemosensory receptors Tlps (Tlp1, Tlp3, Tlp4, Tlp6-8, Tlp10 and CetB), the methyltransferase CheR and the methylesterase CheB, the histidine kinase CheA, the scaffolds proteins CheW/V, the phosphatase CheZ, and the response regulator CheY, that ultimately acts on the flagellar motor to switch rotation either clockwise or counterclockwise (Bren and Eisenbach, 2000; Korolik, 2019), were identified. In a proteomic study, our group observed that Tlp and CheW proteins of *C. fetus* subsp. *venerealis* are significantly upregulated during infection of the genital tract of heifers (Stynen, 2009; Stynen et al., 2009). These genomic and proteomic evidences point that *C. fetus* probably use chemotaxis to reach particular milieu and to the possible participation of chemotaxis in host infection by *C. fetus.*

Since information on the *C. fetus* chemotaxis to substances and structures of bovine genital tract can help to understand and elucidate events related to tissue tropism, niche adaptation and pathogenesis of BGC, we investigated the chemotactic response of *C. fetus* subsp. *venerealis* and *C. fetus* subsp. *fetus* towards cervical mucus, bovine placenta and some of reported substances and ion of bovine cervical mucus and bovine placenta.

## Material and methods

### Bacterial strains and growth conditions

Four *C. fetus* strains were used in this study (Table 1): the type strain of the *C. fetus* subsp. *venerealis*, ATCC 19438^T^ (NCTC 10354^T^); the type strain of the species *C. fetus, C. fetus* subsp. *fetus* ATCC 27374^T^ (NCTC 10842^T^); the host-passaged strain *C. fetus* subsp. *venerealis* P3 (P3) (Stynen, 2009; Haas et al., 2019) and isolate *C. fetus* subsp. *fetus* EV-5 (EV-5) (Leite, 1977). The P3 strain was the *C. fetus* subsp. *venerealis* ATCC 19438^T^ strain recovered from cervical mucus after three serial passages in virgin heifers (Stynen, 2009; Haas et al., 2019).

**Table 1:** *Campylobacter fetus* strains used in chemotaxis assays.

**Strains**	**Subspecies**	**Origin**	**Reference**
ATCC) 19438^T^	*venerealis*	bovine cervical mucus	ATCC
P3*	*venerealis*	bovine cervical mucus	Stynen, 2009; Haas et al., 2019
ATCC 27374^T^	*fetus*	brain of sheep fetus	ATCC
EV-5	*fetus*	aborted bovine fetus	Leite, 1977

ATCC - American Type Culture Collection. * P3 – It is a *C. fetus* subsp. *venerealis* reference strain (ATCC 19438^T^ = NCTC 10354^T^), recovered after three serial passages in virgin heifers (Stynen, 2009; Haas et al., 2019).

Stock cultures in thioglycolate broth containing 20% glycerol at -80 °C were inoculated in blood agar (Brain Heart Infusion (BHI) (Merck, Germany), 1.5% of bacteriological agar (Himedia, India) and 5% defibrinated horse blood) at 37 ºC under microaerophilic conditions (5% O_2_, 10% CO_2_ and 85% N_2_) for 36 hours and subcultured, under the same conditions, twice prior to the chemotaxis assays. To avoid potential alterations due to laboratory passage, P3 strain was subcultured no more than three times. The purity of the cultures was routinely checked by visualizing the morphology of the colonies and, microscopically, by fuchsin staining.

### Cervical mucus and placenta extract and sample design

All biological material and tissues were collected under the Brazilian legislation on animal experimentation (Brasil, 2016) from animals in an abattoir under federal inspection service (SIF).

The placentas were obtained from three pregnant bovine uteruses with estimated pregnancy age of 110, 120 and 140 days, according to the crown-rump (CR) lengths of each fetus, 21, 25 and 33 cm, respectively, based on the methodology of Evans and Sack (1973). The mean age of the fetuses used was therefore 123.33 days, which corresponds to a mean age of 4.11 months of pregnancy. The time was selected since in the cow it is the period in which the majority of abortions by *C. fetus* occurs (Mshelia et al., 2007; Silveira et al., 2018). Intercotiledonary chorioallantoic membrane and fetal cotyledons were placed in phosphate buffered saline (PBS) (0.01 M, pH 7.0, all from Merck, Germany) (1:2) (w/v), macerated aseptically, and centrifuged at 1000 x *g* for 5 minutes at 4 ºC temperature to remove large tissue fragments. The resultant supernatants were collected and mixed (pool) for use in chemotaxis assays.

Mucus was obtained from of the cervicovaginal region from three cows in stage I of estrous cycle (days 1 to 4 of the estrous cycle). The stage of the estrous cycle was estimated according to the methodology of Ireland et al. (1980). Mucus samples were diluted in PBS (1:2) (w/v) and mixed (pool) for use in chemotaxis assays. Mucus and placenta samples were confirmed free of *C. fetus* by multiplex PCR (Hum et al., 1997) before being used in the chemotaxis assays.

### Substances and ion

Substances and ion from the metabolite class of previously reported components of bovine mucus and placenta metabolome (Ware, 1980; Igwebuike, 2006; Will et al., 2010; Dolgorsuren et al., 2017; Tríbulo et al., 2019) and that simultaneously combine energy- chemotactic potential were selected for investigation. In particular, the amino acids L– aspartate, L–glutamate and L-serine, the organic acids fumarate, pyruvate and succinate, the ion ferrous iron, the sugars meso-erythritol and L-fucose and the hormones bovine placental lactogen, 17β-estradiol, and progesterone were tested. The tested concentration ranges of these substances and ion, and their preparations, were based on previous studies of *Campylobacter* spp. growth and chemotaxis (Walsh et al., 1973; Ware, 1980; Hugdahl et al., 1988; Hazeleger et al., 1998; Vegge et al., 2009; Burrough et al., 2012) and in the concentrations reported in genital tract of the bovine female (Pope et al., 1982; Henricks et al., 1983; Inaba et al., 1983; Elhassan et al., 2001; Alvarez-Oxiley et al., 2007; Dolgorsuren et al., 2017), to mimic the physiological range found by the bacteria in the host. Information on all tested substances and ion is listed in Table 2. Amino acids, organic acids, L-fucose, ferrous iron, meso- erythritol and deoxycholic acid were prepared in PBS (Walsh et al., 1973; Hugdahl et al., 1988; Vegge et al., 2009), bovine placental lactogen was diluted in ultrapure water (Alvarez-Oxiley et al., 2007) while 17β-estradiol and progesterone were dissolved in dimethyl sulfoxide (DMSO) (Sigma-Aldrich, USA) and then diluted to desired concentrations in PBS (Burrough et al., 2012). All substances were sterilized by filtration on 0.45 µm filter (Merck, Germany) before their use.

**Table 2:** Substances, ion and concentrations tested in chemotaxis assays of *Campylobacter fetus*.

**Chemical tested**	**Concentrations tested**	**Diluent**	**Source**
Bovine cervical mucus*	1:2 w/v	PBS	Cows in estrus
Bovine intercotiledonary chorioallantoic membrane extract**	1:2 w/v	PBS	Bovine placenta
Bovine fetal cotyledons extract**	1:2 w/v	PBS	Bovine placenta
Substances and ion			
L-aspartate	0.01, 0.1 and 1M	PBS	Sigma-Aldrich1
L-glutamate	0.01, 0.1 and 1M	PBS	Sigma-Aldrich
L-serine	0.01, 0.1 and 1M	PBS	Sigma-Aldrich
Fumarate (sodium)	0.01, 0.1 and 1M	PBS	Sigma-Aldrich
Pyruvate (sodium)	0.01, 0.1 and 1M	PBS	Sigma-Aldrich
Succinate (sodium)	0.01, 0.1 and 1M	PBS	Sigma-Aldrich
Ferrous iron (sulphate)	0.01, 0.1 and 1M	PBS	Merck2
Meso-erythritol	0.01, 0.1 and 1M	PBS	Sigma-Aldrich
Bovine placental lactogen	0.05, 0.5, 5 and 50ng/mL	H_2_O3	ProspecBio4
17β-estradiol	0.05, 0.5, 5 and 50ng/mL	DMSO/PBS5	Sigma-Aldrich
Progesterone	0.05, 0.5, 5, 25, 50, 100 and 200ng/mL	DMSO/PBS	Sigma-Aldrich
Controls			
Deoxycholic acid (chemorepellent)	0.1M	PBS	Merck
PBS pH 7.0 (nonchemotactic)	0.01M	PBS	-
L-fucose (chemoattractant)	0.1M	PBS	Sigma-Aldrich

1 - Sigma-Aldrich, USA; 2 – Merck, Germany; 3 – Ultrapure water, USA; 4 - ProspecBio; 5 – DMSO, Dimethyl sulfoxide/ PBS, phosphate-buffered saline. *Obtained from of the cervicovaginal region from three cows in stage I of estrous cycle (days 1 to 4 of the estrous cycle). **Obtained from bovine pregnant uterus at gestational age between 110 to 140 days.

### Chemotaxis assays

Chemotactic assays were performed using the disk method on soft agar (Vegge et al., 2009) modified by Tareen et al., (2010) as follows. *C. fetus* strains were grown on blood agar (Hazeleger et al., 1998; Tareen et al., 2010; Burrough et al., 2012; Elgamoudi et al., 2018) at 37 ºC under microaerophilic conditions for 36 hours, suspended in PBS, adjusted spectrophotometrically (OD_600_) to approximately 8 x 10^9^ viable CFU/mL and after mixed (1:2) (v/v) with tempered (42 ºC) soft agar (0.8% bacteriological agar; Himedia, India) (Tareen et al., 2010) to obtain the test condition of 4 x 10^9^ viable CFU/mL in PBS-soft (0.4%) agar (Vegge et al., 2009). The number of viable bacteria in each bacterial suspension measured by turbidimetry was confirmed, retrospectively, by the drop counting method (Miles et al., 1938). This method was previously validated for counting *C. fetus* (Haas et al., 2019).

**Figure 1:**
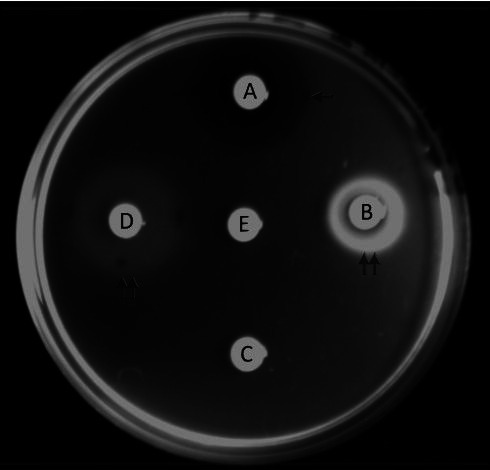
Chemotaxis assays with *Campylobacter fetus* subsp. *venerealis* P3 strain mixed in PBS-soft (0.4%) agar. Filter discs adsorbed with 50 µL of investigated substances and ion were placed on the semi-solidified bacterial suspension, and migration zones (halos) were measured after 4 h of incubation under microaerophilic conditions. (A) 0.1 M deoxycholic acid (chemorepellent; around the disc is a clear zone surrounded by a ring of bacteria that were driven away); (B) 0.1 M ferrous iron (chemoattractant; bacterial accumulation around the disc); (C) progesterone 50 ng/mL (nonchemotactic; no response is observed); (D) L - fucose 0.1 M (chemoattractant); (E) PBS 0.01 M (nonchemotactic control). Arrows point to border of each zone of bacterial accumulation (double arrows) or repulsion (single arrows). *C. fetus* subsp. *venerealis* P3 is a *C. fetus* subsp. *venerealis* reference strain (ATCC 19438^T^= NCTC 10354^T^), recovered after three serial passages in virgin heifers (Stynen, 2009; Haas et al., 2019).

Afterwards, 12 mL of the bacterial soft agar suspension was poured into a 9 cm – diameter Petri dish. Then, sterile filter discs (diameter = 6 mm) (Laborclin, Brazil), soaked with 50 µL of the test substance (Table 2), were placed on the semi-solidified agar (Vegge et al., 2009; Tareen et al., 2010). PBS (0.01 M, pH 7.0), L-fucose (0.1 M) and deoxycholic acid (0.1 M) were used as nonchemotactic, chemoattractant and chemorepellent controls, respectively, based on studies of chemotaxis of *C. jejuni* (Hugdahl et al., 1988; Vegge et al., 2009; Tareen et al., 2010; Dwivedi et al., 2016) and results of a pre-experiment carried with *C. fetus* strains (data not shown). Following 4 hours of incubation at 37 °C under microaerophilic conditions, chemotactic activity was examined over an indirect light source. Bacterial accumulations or clearing zones around a disc were interpreted as zones of attraction toward chemoattractant or repulsion from chemorepellent, respectively (Vegge et al., 2009; Tareen et al., 2010). (Figure1). The diameter of chemotaxis halos was measured in millimeters (mm). The absence of both, accumulation or repulsion in the region around a disc, was interpreted as no response and the substance was classified as nonchemotactic substance (Hugdahl et al., 1988; Vegge et al., 2009; Tareen et al., 2010). (Figure 1). The chemotaxis assays were performed twice for each strain and each concentration of chemical tested (Hugdahl et al., 1988).

### Statistical analysis

The statistical analysis and graphs were performed using the R software (R version 4.0.3, R Development Core Team, New Zealand) (R Core Team, 2020). For the comparisons among bacterial strains in cervical mucus, intercotiledonary chorioallantoic membrane and fetal cotyledons ANOVA was used, followed by Tukey test (Zar, 1996). The same analysis was also used for the comparisons among bacterial strains and concentrations for the same chemical substance. Besides, the different concentrations of the chemical substance were analyzed by linear regression (Zar, 1996). The differences were considered statistically significant when P < 0.05.

## Results

**Figure 2:**
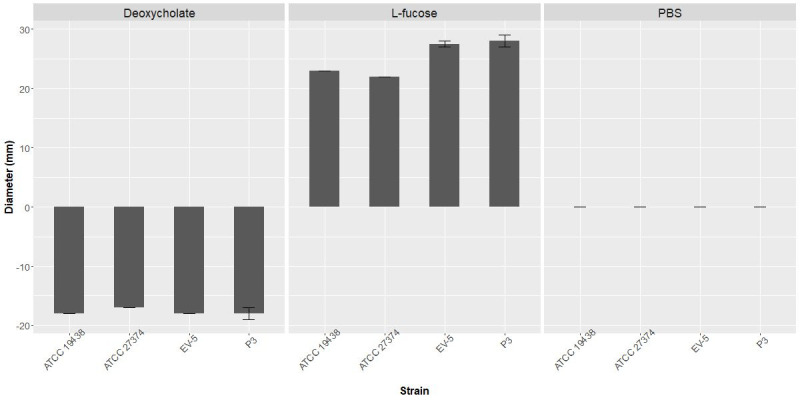
Chemotaxis of *Campylobacter fetus* by deoxycholic acid (0.1 M), L-fucose (0.1 M) and PBS (0.01 M, pH 7.0), used as chemorepellent, chemoattractant and nonchemotactic controls, respectively. The columns show halo diameter size (in millimeters) of chemotaxis. ATCC 19438^T^ – *C. fetus* subsp. *venerealis* reference strain; P3 - *C. fetus* subsp. *venerealis* reference strain (ATCC 19438^T^ = NCTC 10354^T^) recovered after three serial passages in virgin heifers (Stynen, 2009; Haas et al., 2019); ATCC 27374T – *C. fetus* subsp. *fetus* reference strain; EV-5 – *C. fetus* subsp. *fetus* strain isolated of bovine abortion (Leite, 1977).

The controls L-fucose, deoxycholic acid and PBS were attractant, repellant and nonchemotactic, respectively, as expected to all *C. fetus* strains tested (Figure 2).

**Figure 3:**
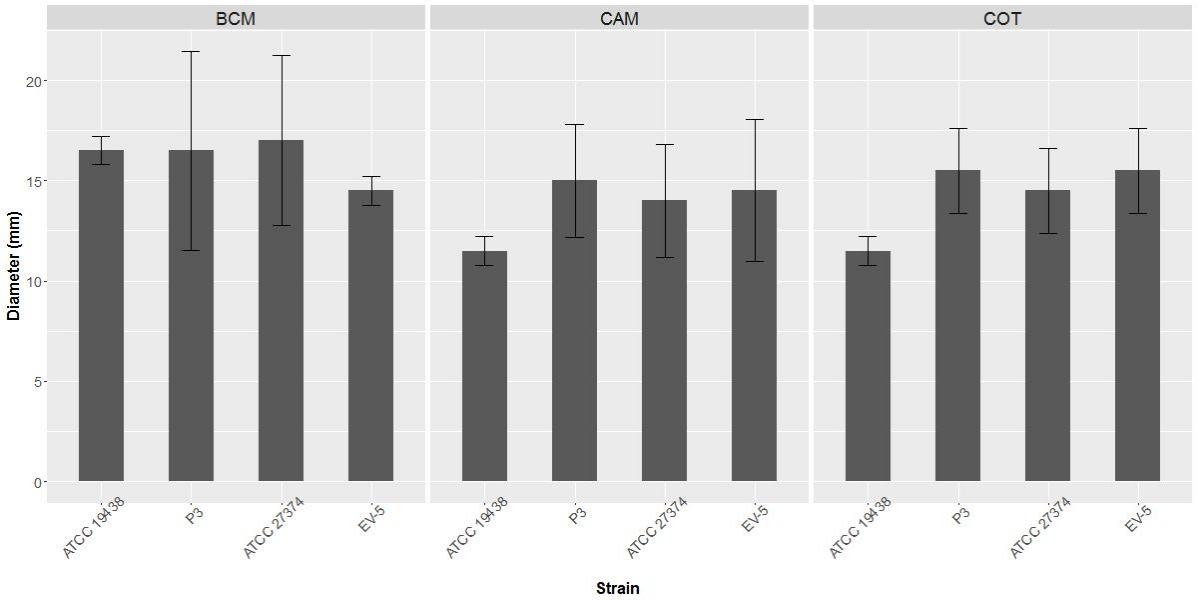
Chemotaxis of *Campylobacter fetus* by bovine cervical mucus (BCM), intercotiledonary chorioallantoic membrane (CAM) and bovine cotyledon (COT) extract. The columns show halo diameter size (in millimeters) of chemoattraction. BCM was obtained of three cows in stage I of estrous cycle (days 1 to 4 of the estrous cycle) and diluted in PBS (1:2) (w/v). The placentas were obtained from three pregnant cows with gestational age between 110 to 140 days and macerated in PBS (1:2) (w/v). ATCC 19438^T^ – *C. fetus* subsp. *venerealis* reference strain; P3 -*C. fetus* subsp. *venerealis* reference strain (ATCC 19438^T^ = NCTC 10354^T^), recovered after three serial passages in virgin heifers (Stynen, 2009; Haas et al., 2019); ATCC 27374^T^ – *C. fetus* subsp. *fetus* reference strain; EV-5 – *C. fetus* subsp. *fetus* strain isolated from bovine abortion (Leite, 1977). Bars show standard error. There was no statistical difference among the strains for the mucus nor the placental extracts.

Bovine cervical mucus was chemoattractant to all *C. fetus* strains evaluated, and no statistical difference was observed among the tested strains (Figure 3).

Bovine intercotiledonary chorioallantoic membrane and fetal cotyledons extracts were chemoattractant to *C. fetus* subsp. *venerealis* and *C. fetus* subsp. *fetus*, however, no statistical differences among the tested strains were observed (Figure 3).

**Figure 4:**
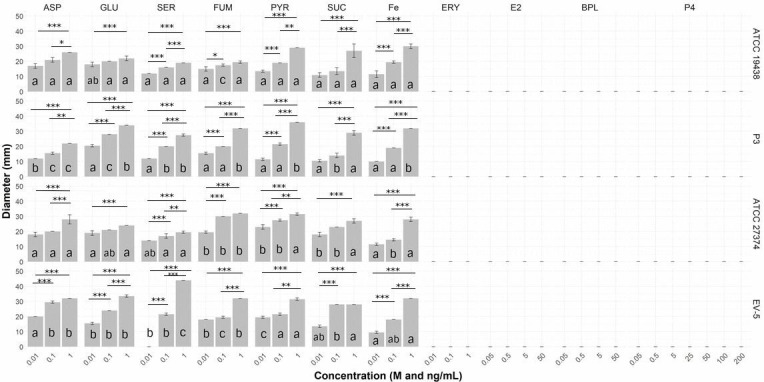
Chemotaxis behavior of *Campylobacter fetus* to reported substances and ions of bovine cervical mucus and substances produced by bovine placenta. The columns show halo diameter size (in millimeters) of chemoattraction. L-aspartate (ASP), L-glutamate (GLU), L-serine (SER), fumarate (FUM), pyruvate (PYR), succinate (SUC) ferrous iron (Fe), meso-erythritol (ERY) concentrations is in molar (M). The 17 β-estradiol (E2), bovine placental lactogen (BPL) and progesterone (P4) concentrations is in ng/mL. ATCC 19438^T^ – *C. fetus* subsp. *venerealis* reference strain; P3 - *C. fetus* subsp. *venerealis* reference strain (ATCC 19438^T^ = NCTC 10354^T^) recovered after three serial passages in virgin heifers (Stynen, 2009; Haas et al., 2019); ATCC 27374^T^ – *C. fetus* subsp. *fetus* reference strain; EV-5 – *C. fetus* subsp. *fetus* strain isolated of bovine abortion (Leite, 1977). Bars show standard error. *P < 0.05; **P < 0.01; ***P < 0.001 indicate levels of significant statistical difference between concentrations in the same substance in the same strain. Different lowercase letters within the same substance and concentration reflect significant statistical difference among bacterial strains.

**Figure 5:**
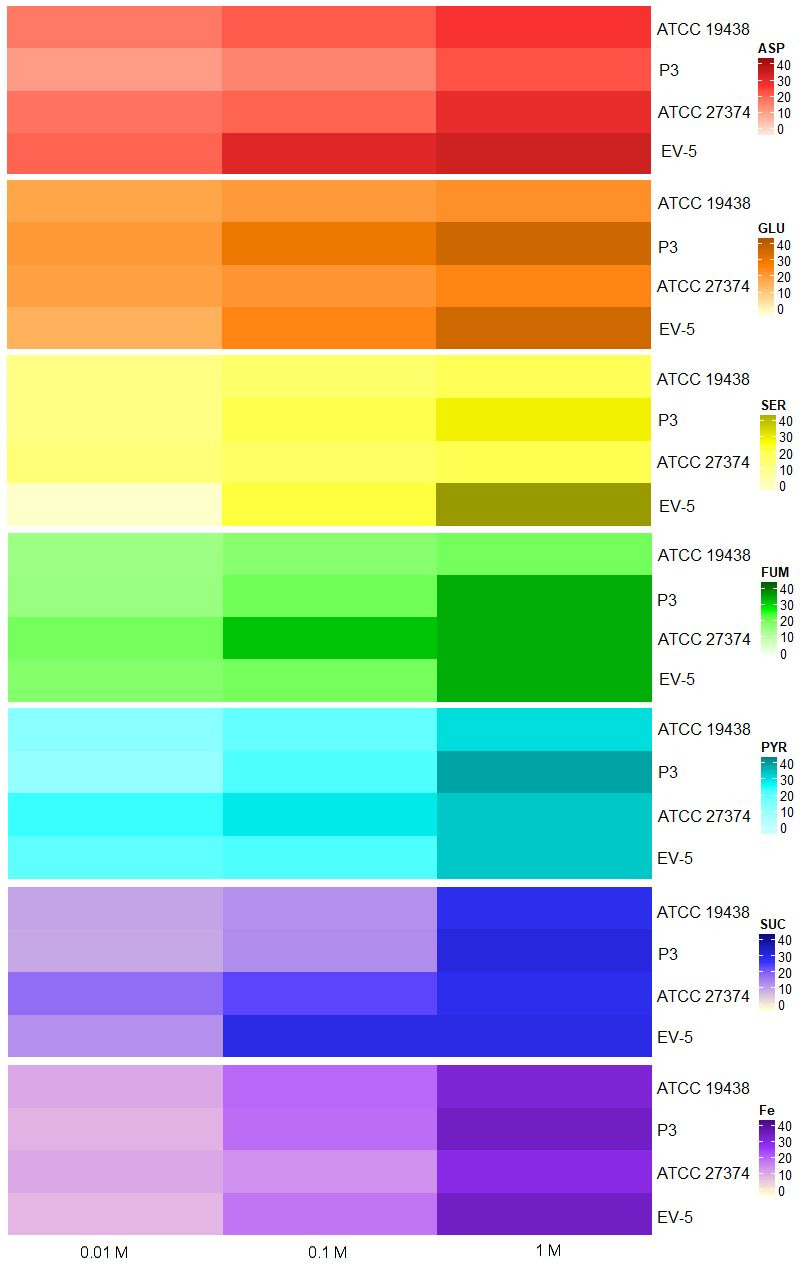
Heatmap of the chemoattractive substances and ion for the investigated *Campylobacter fetus* strains. ASP - L-aspartate, GLU - L-glutamate, SER - L-serine, FUM – fumarate, PYR - pyruvate, SUC - succinate, Fe - ferrous iron. ATCC 19438^T^ – *C. fetus* subsp. *venerealis* reference strain; P3 - *C. fetus* subsp. *venerealis* reference strain (ATCC 19438^T^ = NCTC 10354^T^) recovered after three serial passages in virgin heifers (Stynen, 2009; Haas et al., 2019); ATCC 27374^T^ – *C. fetus* subsp. *fetus* reference strain; EV-5 – *C. fetus* subsp. *fetus* strain isolated of bovine abortion (Leite, 1977). Regression analysis revealed that the increase in chemoattraction was significantly higher as concentration increases. The R^2^, the variance explained by model, was greater than 95% for all attractive substances and ion, except for L-serine, which still presented a high R^2^ (86%), but lower than the others.

The substances and ion L-aspartate, L-glutamate, L-serine, pyruvate, succinate, fumarate, and ferrous iron, were chemoattractant to all *C. fetus* strains evaluated, being the halos significantly larger and visually denser in higher concentrations of the tested substances (Figure 4 and 5). Regression analysis revealed that the increase in chemoattraction was significantly higher as concentration increases. The R^2^, the variance explained by model, was greater than 95% for all attractive substances and ion, except for L-serine, which still presented a high R^2^ (86%), but lower than the others.

The chemoattraction haloes of P3 strain for L-glutamate, L-serine, fumarate, pyruvate, and ferrous iron was significantly higher compared with that observed for *C. fetus* subsp. *venerealis* ATCC 19438^T^ parental strain. In contrast, *C. fetus* subsp. *venerealis* ATCC 19438^T^ strain showed greater chemoattraction halos for L-aspartate than P3 strain. The chemoattraction of the EV-5 strain for L- aspartate, L-glutamate, L-serine was significantly higher when compared with that observed for *C. fetus* subsp. *fetus* ATCC 27374^T^ strain. *C. fetus* subsp. *fetus* ATCC 27374^T^ strain showed greater chemoattraction halos for fumarate and pyruvate than the three strains of bovine origin, *C. fetus* subsp. *venerealis* ATCC 19438^T^, *C. fetus* subsp. *venerealis* P3 and *C. fetus* subsp. *fetus* EV-5.

Meso-erythritol, bovine placental lactogen, 17β-estradiol and progesterone were nonchemotactic to all *C. fetus* strains investigated.

## Discussion

Chemotaxis is cell movement in response to chemical cues employed by bacterial pathogens to migrate towards environments that are better for growth. Consequently contributes to these microorganisms to reach their preferred host niches, being an important subject of tissue tropism (Porter et al., 2011; Johnson and Ottemann, 2018; Matilla and Krell, 2018; Korolik, 2019; Yang and Ottemann, 2019). In the present study, we report the chemotactic behavior of reproductive pathogen *C. fetus* towards cervical mucus, bovine placenta and some reported substances and ion of bovine cervical mucus and bovine placenta, which brings an insight on the use of chemotaxis by *C. fetus* to reach their preferred colonization niches and the tissue tropism of this bacterium.

Penetration and survive in mucus layer, guided by chemotaxis, is an essential step during the colonization of mucous surfaces by motile bacteria, favoring the establishment of bacterial populations in this environment (Alemka et al., 2012; Yang and Ottemann, 2019). In this context, attraction to mucus during the estrus, period in which the infection occurs, is a very relevant finding to the *C. fetus* pathogenesis in genital tract of the bovine female, since the bacterium is able to remain in the genital tract by successfully colonizing the mucus layer (Ware, 1980; Balzan et al., 2020). This ability of campylobacters to colonize the mucus is facilitated by its spiral cell shape that creates a corkscrew-like rotation, by the swimming behavior (flagellar motility) (Blaser et al., 2008; Balzan et al., 2020) and probably by chemotaxis, which guides the environmental navigation in this viscous layer. The biological significance of mucus chemotaxis may be that it increases nutrient acquisition efficiency and enhance the known ability of *C. fetus* to metabolize amino acids and organic acids within the mucus (Ware, 1980; Blaser et al., 2008), favoring bacterial multiplication in the genital tract of cows. In fact, in the presence of bovine cervical mucus, the respiratory activity of *C. fetus* is increased, evidenced by higher the rates of oxygen uptake (Ware, 1980), suggesting the use of these substrates as carbon source. This assumption corroborates the findings for the reported components of the mucus investigated, where it was observed that the chemical constituents that elicited a positive chemotactic response (Figure 3) are the main sources of energy for *C. fetus*. They are metabolic substrates (L- aspartate, L-glutamate, and L-serine), electron donors (pyruvate and succinate) and electron acceptors (fumarate) (Blaser et al., 2008). These evidences show that *C. fetus* swims towards substrates for which is possible to obtain energy from oxidative phosphorylation and tricarboxylic acid cycle. Positive chemotaxis for these amino acids and organic acids suggests that these substances and ion may be involved in chemotaxis for cervical mucus, since they are the main energy sources for *C. fetus* and are among the most abundant components of bovine cervical mucus (Ware, 1980; Elhassan et al., 2001; Tríbulo et al., 2019). Indeed, positive chemotactic response to amino acids and organic acids may facilitate bacterial penetration on mucus layer, favoring the survival and colonization of mucosal surfaces.

*C. fetus* attraction to L-fucose is a particularly interesting observation and may have direct relevance *in vivo,* since fucose is a terminal sugar in the mucins of the genital tract of the cow (Pluta et al., 2011) and serves as binding target for campylobacters to the mucosal epithelium (Dwivedi et al., 2016). This ability to sense fucose could drives the microorganism towards a chemical gradient of fucose in the genital environment and mediate the binding of *C. fetus* to fucosylated structures from epithelium, which are important target sites for colonization. Other mucin glycans, such galactose and N-acetylgalactosamine, have been suggested as potential binding sites for *C. fetus* subsp. *venerealis* in the uterus of heifers (Cipolla et al., 1998).

In addition, this L-fucose taxis may also be associated to use of sugar as source of carbon, as it occurs with *C. jejuni* strains that have the genetic locus (*fuc* locus) that encode pathways for fucose uptake/metabolism and are able to catabolize fucose (Dwivedi et al., 2016). In*C. fetus*, the genes encoding the fucose transporter FucP and the enzymes required to degrade fucose, are also present, predicting that *C. fetus* may be able to metabolize L-fucose (Stynen et al., 2011; Oliveira et al., 2016), however, the operability of the pathway and the fucose metabolism of *C. fetus* were not evaluated.

Our results regarding ferrous iron clearly showed that this substrate is a powerful attractant to *C. fetus,* which could be related to the central role that the iron has in essential metabolic processes. Iron is a cofactor for proteins involved of cellular metabolism, enzyme catalysis, and sensing extracellular and intracellular signals (Chandrashekhar et al., 2018). The sensing and navigation of *C. fetus* towards ferrous iron may be mediated by FeoAB system, a predicted system in the genome of *C. fetus* subsp. *venerealis*, including ATCC 19438^T^ strain (Stynen et al., 2011) and believed to carry the environmental ferrous iron across the outer membrane by porins and through the cytoplasmic membrane, by transporter proteins FeoA and FeoB (Naikare et al., 2006; Blaser et al., 2008). The movement towards the ferrous iron, as observed for amino acids, organic acids and fucose, probably also has an important role in the pathogenesis of BGC, because can enhance the uptake and use of this essential nutrient. The uptake of ferrous iron in large amounts is a crucial event for the success of infection and is a determinant of colonization for *C. jejuni* (Naikare et al., 2006; Chandrashekhar et al., 2018). Interestingly, the chemoattraction zones were larger in higher concentrations of ferrous iron, amino acids, and organic acids (Figure 3), clearly showing that *C. fetus* navigates further towards environments with higher concentrations of these substances.

Differences are reported when comparing host-passaged strains with laboratory-adapted reference strains (Stynen, 2009; Haas et al., 2019) and it was also observed in our past and present studies. For example, heifer-passaged *C. fetus* subsp. *venerealis* induced higher expression of CXCL8 chemokine by HeLa cells than the parental *C. fetus* subsp. *venerealis* reference strain ATCC 19438^T^ (Haas et al., 2019). The significantly greater attraction of the *C. fetus* subsp. *venerealis* P3 strain to several of the investigated substances (glutamate, serine, fumarate, pyruvate, and ferrous iron) compared to *C. fetus* subsp. *venerealis* reference parental strain ATCC 19438^T^ suggests that the passage in the host increased chemotactic capacity of *C. fetus* subsp. *venerealis* P3 strain and that this group of substances can be very important during host infection. In contrast, the reduced chemotaxis of the *C. fetus* subsp. *venerealis* P3 strain when compared with the reference *C. fetus* subsp. *venerealis* strain ATCC 19438^T^ suggests that aspartate would be less important during *in vivo* infection. The globally reduced chemotactic profile of *C. fetus* subsp. *venerealis* laboratory-adapted ATCC 19438^T^ strain probably results from laboratory subculture over time, that is known to cause a general loss of virulence, including decrease chemotactic motility related genes/proteins expression in campylobacters (Stynen, 2009; Cooper et al., 2013; King et al., 2013). Thus, the higher chemotactic activity of *C. fetus* subsp. *fetus* EV-5 strain for aspartate, glutamate, and serine, when compared with the *C. fetus* subsp. *fetus* ATCC 27374^T^ reference strain may be due to fact that the EV-5 strain has fewer subcultures compared with the ATCC 27374^T^ strain. We should also consider the possible influence of the differences in origin of the strains, as *C. fetus* subsp. *fetus* EV-5 is a bovine isolate while *C. fetus* subsp. *fetus* ATCC 27374^T^ is an ovine isolate. *C. fetus* subsp. *fetus* ATCC 27374^T^ strain showed significantly higher chemotaxis to organic acids (fumarate, pyruvate, and succinate) when compared to the three strains of bovine origin, *C. fetus* subsp. *fetus* EV-5, *C. fetus* subsp. *venerealis* ATCC 19438^T^ and *C. fetus* subsp. *venerealis* P3.

Tropism for placental tissues is a frequent phenomenon in *C. fetus* infection (Vargas et al., 2002; Sahin et al., 2017; Farace et al., 2019) and an important step in the pathogenesis of BGC, since it enhances the access of large numbers of bacterial cells to the placenta and thereby influences the course of infection. Our *in vitro* findings indicate that *C. fetus* uses chemotaxis *in vivo* to reach the placenta and infects the tissue with large numbers of bacterial cells, which may be important for rapid bacterial establishment and to lead to abortion. This dynamics of bacterial navigation through the genital tract can be also influenced by the animal immune status, being facilitated and accelerated in non-immune animals or, on the other hand, made it more difficult and delayed due to the action of the immune response to previously *C. fetus* – infected animals. Therefore, our results of *C. fetus* attraction to intercotiledonary chorioallantoic membrane and fetal cotyledon from the second gestational trimester could also partially explain why the majority of abortions due to *C. fetus* infection are mostly noted at 4 to 6 months of pregnancy in BGC (Mshelia et al., 2007; Sahin et al., 2017; Silveira et al., 2018).

Our findings also indicate the presence of chemotactic factors for *C. fetus* in bovine placenta. The bovine placental trophoblast is an exuberant producer of erythritol, 17β-estradiol, placental lactogen and, especially, progesterone (Igwebuike, 2006; Nguyen et al., 2012; Letesson et al., 2017). However, in our assays, at physiological level concentrations that simulate the host environment, all of these substances were nonchemotactic for *C. fetus*, as bacteria do not respond chemotatically to these individual substances.

The non-chemotactic behavior of *C. fetus* to erythritol possibly stems from the absence of a catabolic pathway, such as that found in *Brucella abortus*, which allows the metabolism of erythritol and has been implicated in placental tropism by *B. abortus* (Letesson et al., 2017). Like erythritol sugar, failure of placental lactogen, which is produced only during pregnancy by binucleate placenta cells (Alvarez-Oxiley et al., 2007), and estradiol, which is produced in substantial amounts by the trophoblast (Inaba et al., 1983; Nguyen et al., 2012), to stimulate chemotactic response to *C. fetus* suggest that both hormones also did not guide the swimming of *C. fetus* towards the bovine placenta. In addition, this inert behavior by estradiol means that ovarian estradiol from the estrous follicular phase may have no effect on the *C. fetus* taxis during estrus, at which stage infection transmission and vaginal colonization occurs.

The chemotactic effects of progesterone were of particular interest, as bovine high levels of progesterone in the uterus in the luteal phase of the reproductive cycle (Pope et al., 1982) and the increased placental synthesis of progesterone in the second trimester of pregnancy (Nguyen et al., 2012), which could explain, in part, the ascension of *C. fetus* to the uterus and the occurrence of abortions during this period. However, the absence of chemotaxis towards progesterone, at levels that mimic its estrous cycle and pregnancy concentrations, indicate that *C. fetus* is not responding chemotactically to progesterone and suggest that this hormone does not drives the rise of the bacterium to the uterus and placenta.

The fact that the placenta extract elicits chemotactic response in *C. fetus*, but reported hormones (placental lactogen, 17β-estradiol and progesterone) and erythritol did not, suggests that the attraction *C. fetus* by placental extract could have occurred due to the presence of other placental components, such as amino acids. Amino acids are abundant in this tissue, as previously reported (Dolgorsuren et al., 2017) and were strong chemoattractants to *C. fetus* in the study. In addition, we must also consider that one or more placental components, which were not evaluated in the present study, may be involved in the chemoattraction of *C. fetus* by bovine placenta.

## Conclusion

In conclusion, *C. fetus* exhibits chemotaxis towards bovine cervical mucus and placenta extracts as well as some substances and ion reported in these materials, such as amino acids, ferrous iron, fucose and organic acids. This chemotactic behavior may guide the *C. fetus* navigation in host and be an important subject of tropism for placenta and bovine female genital tract.
